# Kynurenine pathway modulation reverses the experimental autoimmune encephalomyelitis mouse disease progression

**DOI:** 10.1186/s12974-020-01844-y

**Published:** 2020-06-06

**Authors:** Gayathri Sundaram, Chai K. Lim, Bruce J. Brew, Gilles J. Guillemin

**Affiliations:** 1 Peter Duncan Neurosciences Research Unit, St Vincent’s Centre for Applied Medical Research, Sydney, NSW 2010 Australia; 2 St Vincent’s Clinical School, Faculty of Medicine, University of New South Wales, Sydney, NSW 2052 Australia; 3 Faculty of Medicine, Health and Human Sciences, Macquarie University, Sydney, NSW 2109 Australia; 4 Department of Neurology, St Vincent’s Hospital, Sydney, NSW 2010 Australia; 5 Neuroinflammation Group, Faculty of Medicine, Health and Human Sciences, Macquarie University, Sydney, NSW 2109 Australia

**Keywords:** Multiple sclerosis, Tryptophan, Kynurenine pathway, Quinolinic acid, Neuroinflammation, Neurodegeneration

## Abstract

**Background:**

Multiple sclerosis (MS) is a chronic immune-mediated disorder of the central nervous system characterized by demyelination, neuroinflammation, and neurodegeneration. Activation of the kynurenine pathway (KP) results from acute and chronic neuroinflammation leading to both immune suppression and neurotoxicity. However, the exact effects of KP metabolites and changes in neurodegenerative diseases over time are not fully understood. Studies, including those in MS models, have reported that short-term KP activation is beneficial through immune tolerance. However, the effects of long-term KP activation are poorly understood. We hypothesized that such chronic activation is responsible for the neurodegeneration in MS, and further, modulating the KP in EAE-induced mice could significantly decrease the EAE disease severity.

**Methods:**

We biochemically altered the KP at different stages of the disease in experimental allergic encephalomyelitis (EAE) mouse model of MS and at two different enzymatic levels of the KP (IDO-1 (indoleamine 2,3 dioxygenase)) and KMO (kynurenine monooxygenase). CNS tissue and blood samples were analyzed longitudinally using GCMS, HPLC, IHC, and RT-PCR.

**Results:**

We showed that the KP was steadily upregulated correlating with disease severity and associated with a shift towards increasing concentrations of the KP metabolite quinolinic acid, a neuro- and gliotoxin. KP modulation by inhibition of IDO-1 with 1-methyl tryptophan (1-MT) was dependent on the timing of treatment at various stages of EAE. IDO-1 inhibition at EAE score 2 led to significantly higher numbers of FoxP3 cells (*p* < 0.001) in the spleen than earlier IDO-1 inhibition (prophylactic 1-MT treatment group (*p* < 0.001)), 1-MT treatment after EAE induction (EAE score 0; *p* < 0.001), and 1-MT treatment at EAE score of 1 (*p* < 0.05). Significant improvement of disease severity was observed in EAE mice treated with 1-MT at EAE score 2 compared to the untreated group (*p* < 0.05). KP modulation by KMO inhibition with Ro 61-8048 led to significantly greater numbers of Foxp3 cells (*p* < 0.05) in Ro 61-8048 treated mice and even more significant amelioration of EAE disease compared to the 1-MT treatment groups.

**Conclusions:**

These results provide a new mechanistic link between neuroinflammation and neurodegeneration and point to KP modulation at the KMO level to preserve immune tolerance and limit neurodegeneration in EAE. They provide the foundation for new clinical trials for MS.

## Background

**Fig. 1:**
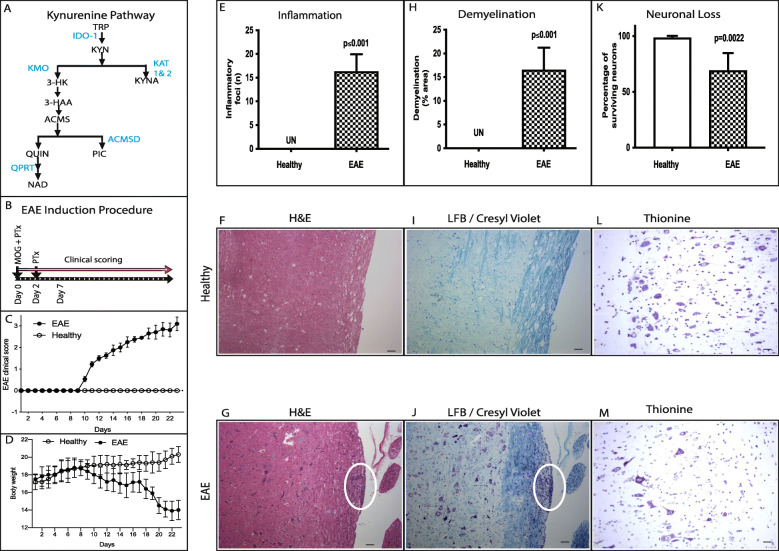
Experimental autoimmune encephalomyelitis (EAE). **a** Schematic illustration of kynurenine pathway (KP). KP is depicted as metabolites in black, enzymes in blue. **b** Experimental procedure to induce experimental autoimmune encephalomyelitis (EAE) in C57BL/6 mice with MOG_35-55_ peptide (myelin oligodendroglial protein) and pertussis toxin (PTx). **c** Mean daily clinical score in the EAE (*n* = 18) and healthy mice (*n* = 15). **d** Body weight of EAE and healthy mice was determined (mean ± SD). **e**–**i** Inflammation, demyelination, and neuronal loss in EAE-induced mice spinal cord. **e** The inflammatory foci (mean ± SD) counted in H&E stained axonal sections of EAE and healthy control. **f** and **g** Representative histological spinal cord sections of healthy (**f**) and EAE mice (**g**) stained with H&E. **h** Demyelination (% area) without inflammation detected by Luxol Fast Blue (LFB)/cresyl violet staining. **i** and **j** Representative histological spinal cord sections of healthy (**i**) and EAE mice (**j**) stained with LFB/cresyl violet. **k** Surviving neurons stained for Nissl substances using 0.1% thionine and neurons with well-defined nucleolus were counted. **l** and **m** Representative histological spinal cord sections of healthy (**l**) and EAE mice (**m**) stained with thionine for surviving neurons; white oval shows inflammatory foci (**l**, *n* = 6) and demyelinated area (**m**, *n* = 6). Scale bar 10 μm (**f**, **g**, **i**, and **j**), 25 um (**l** and **m**). IDO-1, indoleamine 2,3-dioxygenase; KAT, kynurenine amino transferase; KMO, kynurenine monooxygenase; KYNU, kynureninase; 3HAO, 3-hydroxyanthranilate 3,4- dioxygenase; ACMSD, 2-amino-3-carboxymuconate-semialdehyde decarboxylase; QPRT, quinolinate phosphoribosyl transferase

Multiple sclerosis (MS) is a complex inflammatory and demyelinating disease of the central nervous system (CNS) characterized by the presence of plaques [1]. These are associated with immune activation, especially of the innate immune system [2]. Much of the evidence for MS pathogenesis and its disease modifying therapies comes from studies of the experimental autoimmune encephalomyelitis (EAE) mouse model. Traditionally, the CD4+ T-cell subsets, Th1 and Th17 cells, are considered to be the encephalitogenic T-cells in both MS and EAE mice. These Th1 cells have many effects, but the relevance here is that they increase the secretion of the proinflammatory cytokine and interferon-γ (IFN-γ) [3]. While IFN-γ has pleiotropic effects, it is also one of the most potent activators of the kynurenine pathway (KP). IFN-γ induces indoleamine 2,3-dioxygenase (IDO-1), a tryptophan (TRP)-catabolizing enzyme, which is the first enzyme of the KP (see Fig. 1a). IDO-1-expressing cells exert a profound inhibitory effect on T-cells, which in turn suppress the proinflammatory processes that occur in response to tissue damage [4]. However, KP activation also leads to increasing amounts of 3-hydroxy kynurenine (3-HK), a free radical generator, as well as quinolinic acid (QUIN), a neuro- and gliotoxin acting primarily through the N-methyl-D-aspartate (NMDA) receptor. Kynurenic acid (KYNA), a neuroprotective antagonist of NMDA receptors, is also produced. The relative amounts of these, and their effects in the context of chronic inflammation, are poorly understood.

There are several lines of evidence supporting the presence of activation of the KP in MS and its biological significance. In MS patients, TRP levels are decreased in both plasma and CSF [5]. This is potentially biologically significant as IDO-1 activation leads to decreased inflammation through suppression of T-cell responses and promotion of tolerance [6]. Furthermore, there is increased expression and production of the KP enzymes, kynurenine amino transferases 1 and 2 (KAT 1 and 2), in red blood cells and their resulting metabolite KYNA in the plasma of MS patients [7]. Indeed, the CSF KYNA concentrations are increased during an acute relapse and decreased in the chronic phase [8].

In the long term, MS is often associated with neurodegeneration. Approximately 50% of relapsing remitting MS patients will eventually develop a secondary progressive phase characterized by neurodegeneration without relapses but still associated with neuroinflammation. Presently, it is not clear whether the two processes of neuroinflammation and neurodegeneration are causally linked, or mostly independent. While the short-term role of KP activation appears to be beneficial in suppressing neuroinflammation, long-term KP activation is likely detrimental due to the production of excessive neurotoxic metabolites of the KP. This is supported by our earlier study on MS patients showing a shift in the KP towards more neurotoxic products that correlated with worsening of the disease and the switch to the secondary progressive phase of MS [9]. Given the relationship between the KP and neuroinflammation and neurodegeneration, especially the KP derived toxin QUIN, we hypothesized that activation of the KP in MS provides a new mechanistic link between neuroinflammation and neurodegeneration. To test this, we used a chronic-EAE mouse model to better represent the chronic progressive form of MS due to similarities in disease susceptibility, course, and histopathology. The KP was inhibited at the initial step (IDO-1) and midway at the kynurenine monooxygenase (KMO) step (Fig. 1a). Our hypothesis was that modulating the KP in EAE-induced mice could significantly decrease the EAE disease severity.

We injected 1-methyl-tryptophan (1-MT; IDO-1 inhibitor) and Ro 61-8048 (KMO inhibitor) after the mice had developed EAE at a score of 2. This was to ensure that IDO-1 induction had enough time to trigger immune tolerance and to determine the therapeutic ability of KP modulators to prevent neurodegeneration (as opposed to other studies that had inhibited the KP at an earlier time point before the development of tolerance). The clinical course was measured in conjunction with immunohistochemistry (IHC), qRT-PCR, uHPLC, and GC-MS techniques to quantify the KP metabolites involved in neuroprotection and neurotoxicity. Our results reveal a dynamic interplay between KP metabolites and EAE disease development. They demonstrate that KP activation is a crucial component in MS neuropathogenesis and link neuroinflammation with neurodegeneration. Furthermore, this study demonstrates that altering the KP with specific enzyme inhibitors is likely to have therapeutic potential for MS.

## Materials and methods

### Animals

Female C57BL/6 mice of 6–8 weeks of age and body weight of 16–21 g were used. All animals in this study were obtained from the Australian Bio Resources Ltd, New South Wales. The animals were kept under controlled temperature conditions (22 ± 2 °C), humidity (55 ± 10%), light cycle (12 h light/12 h dark), and air exchange for the duration of the experiment. Experimental research involving biological materials had Garvan/St Vincent’s Animal Ethics Committee approval (AEC No. 08/24). All animal experiments were carried out in accordance with the Australian code of practice for the care and use of animals for scientific purpose (8th edition 2013-NHMRC).

### Reagents

Desiccated, killed mycobacterium tuberculosis H37Ra was obtained from Bioscientific Pty Ltd. Complete Freund’s adjuvant (CFA) was purchased from Sigma-Aldrich (MO, USA). Myelin oligodendrocyte glycoprotein (MOG_35-55_; amino acid [10] sequence: H-Met-Glu-Val-Gly-Trp-Tyr-Arg-Ser-Pro-Phe-Ser-Arg-Val-Val-His-Leu-Tyr-Arg-Asn-Gly-Lys-OH) and pertussis toxin were obtained from Sapphire Biosciences. Interchangeable syringes were obtained from Cadence Inc (VA, USA). 1-Methyl-DL-tryptophan (Sigma-Aldrich, MO, USA) was diluted in 5N HCl and adjusted to pH 7 before injection [11]. Ro 61-8048 (Tocris, Bristol, UK) was dissolved in DMSO and diluted in sterile 0.9% saline to a concentration of 10 mg/ml, and the pH was adjusted to 7.5 [12].

### Active induction of experimental autoimmune encephalomyelitis in mice

EAE induction was optimized in our laboratory based on an established protocol [13]. Briefly, mice under inhalation anesthesia (4% isofluorane) were immunized with 100 μg of MOG_35-55_ and 1 mg of M. tuberculosis in complete Freund’s adjuvant (Sigma-Aldrich; MO, USA) on day 0. Additionally, mice received 200 ng of pertussis toxin intraperitoneally on days 0 and 2. Clinical signs of EAE were assessed daily as per the approved EAE guidelines; 0, normal; 1, loss of tail tone or hind limb weakness; 2, both loss of tail tone and hind limb weakness; 3, partial hind limb paralysis; 4, both hind limb paralysis; and 5, moribund state [14]. Where appropriate, mice were given in-between scores (i.e., 0.5, 1.5, 2.5, 3.5) to record a complete disease course.

The mice developed clinically induced EAE 9–14 days after immunization. To analyze the KP metabolism in EAE disease progression, we grouped these mice according to their clinical score: cohort I (EAE score 0.5–1), cohort II (EAE score 1.5–2), and cohort III (EAE score 3–4). After the mice reached the desired EAE clinical score and remained at that score for 2 consecutive days, they were deeply anesthetized to collect blood and CNS tissues for analyzing the KP metabolites at the molecular and protein level. Sham injected EAE mice served as healthy controls.

### Drug treatments

#### 1-Methyl-DL-tryptophan (1-MT; IDO-1 inhibitor) treatment groups

Prophylactic treatment of mice prior to EAE induction with 1-MT on day 0, 1-MT treatment on day 5 after EAE induction (score 0), and 1-MT treatment after the EAE-induced mice reached the score of 1 or 2 and remained in the same score for 2 consecutive days (Fig. 4a). These groups were injected with 1-MT intraperitoneally at a concentration of 100 mg/kg body weight daily for 7 days.

#### Ro 61-8048 treatment

Treatment of EAE-induced mice was initiated when the mice reached the score of 2 and remained at the same score for 2 consecutive days. The KP inhibitor, Ro 61-8048 (KMO inhibitor), was injected intraperitoneally at a concentration of 100 mg/kg daily for 7 days.

Clinical severity was measured based on the approved clinical scale as mentioned above. After the treatment period, plasma and CNS tissues were harvested to analyze KP metabolites. Saline-treated animals were used as vehicle control.

### qPCR

For reverse transcription-PCR experiments, six animals/group were analyzed. Total RNA was extracted from the mouse brain and spinal cord by using RNA isolation kit (Qiagen RNeasy Mini Kit). cDNA was transcribed from up to 1 μg of total RNA using Superscript III VILO cDNA synthesis kit (Invitrogen). A 5 μl sample of cDNA (equivalent to 5 ng RNA) was used per 20 μl reaction mix in each well, with 10 μl of express SYBR green qPCR super mix universal (Invitrogen), and 0.6 μl of each primer (10 μM) to bring to 300 μM final concentration per well. The oligonucleotide sequences of the primers (*IDO-1*, *KAT 1* and *2*, aminocarboxymuconate semialdehyde decarboxylase (*ACMSD*), quinolinate phosphoribosyl transferase (*QPRT*)) used for RT-PCR are obtained from the previous study [15].

For quantitative PCR reactions, the reaction mixture consisted of Invitrogen SYBR Green master mix, 100 nM of each forward and reverse primer, and 5 ng of cDNA sample toped up with nucleic acid-free water. Analysis of the expression of each individual gene was performed in triplicate from samples obtained from 3 to 4 experimental repeats. The SYBR-Green based three-step PCR protocol divided into 3 segments. Segment 1: 50 °C for 2 min followed by 95 °C for 2 min; segment 2: 40 cycles of 95 °C for 15 sec, 60 °C for 2 s; and segment 3: 95 °C for 1 min, then 60 °C for 30 s followed by 95 °C for 30 s using SYBR-Green based primers. Before quantitative PCR assessment, the efficiency of primers for each individual gene was determined by performing quantitative PCR on a serial dilution of positive control samples. The efficiency of the primers was then derived from the slope of the standard curve representing expression levels for serial dilutions of the control samples. The primer efficiencies were then used to determine relative gene expression by comparative cycle time method [16]. Comparative results are presented relative to the housekeeping gene, RPL13.

### Quantification of KP metabolites

A simple extraction procedure using trichloric acid (TCA) was used to deproteinise the CNS samples [9]. For every milligram of tissue, 20 μl of PBS was added and then homogenized in a Precelly’s tissue homogenizer. The homogenized solution was then mixed with equal volume of 10% TCA and then homogenized and centrifuged at 12,000×*g* for 10 min at 4 °C. Supernatants were collected and filtered through 0.45-μm ACRODISC® CR 4-mm PTFE syringe filter (Waters Corporation, MA, USA). The plasma samples were treated in the same manner with 10% TCA but without homogenization. KP metabolites were then analyzed by ultra-high performance liquid chromatography (uHPLC) to detect TRP, KYN, KYNA, and gas chromatography-mass spectrometry (GCMS) to measure QUIN and PIC, as described previously [9].

### NAD^+^/NADH quantification assay

Cellular NAD^+^ and NADH levels were measured using the NAD^+^/NADH-Glo™ kit (Promega) according to the manufacturer’s instructions.

### Histopathological analysis

Histopathological analysis of mice tissue was performed on PFA-fixed sections of lumbar spinal cord. The neuropathological evaluation was performed using hematoxylin-eosin (H&E), Luxol fast blue (LFB), and thionine staining to assess the inflammatory infiltrates, demyelination, and surviving neurons, respectively. The slides were examined under a bright field microscope (Leica DM4000) using a 3CCD camera. Each section displaying the infiltration of mononuclear cells was assigned a score of one inflammation. The number of inflammatory infiltrates was calculated as the mean number of inflammatory infiltrates per spinal cord section. For analysis of demyelination, adjacent serial spinal cord sections were stained with LFB and counter stained with cresyl violet. Tissue damage was marked with ImageJ and expressed as the percentage of lesioned area on the spinal cord section. At least 2 animals per group were analyzed, and 10–18 different rostrocaudal sections were marked per animal. The Nissl staining was used to evaluate the general neuronal morphology. To quantify the Nissl substances (using 0.1% thionine), the cells in the ventral horn of the spinal cord were counted using the Image J software. Neuron counts were performed and restricted to the neurons with a well-defined nucleolus and cell body. Statistical significance was assessed by unpaired Student’s *t*-test.

The immunohistochemical staining was performed on 4% PFA fixed, paraffin embedded spinal cord sections. In brief, the spinal cord sections (5 μm) were treated with 3% H_2_O_2_ for quenching of endogenous peroxidase activity. Sections were then treated with serum-free protein block (Dako, X0909) before incubation with antibody against IDO-1 (Biolegend 122401;), KMO (Abcam; ab83929), quinolinate phosphoribosyl transferase (QPRT) (Abnova; H00023475), and forkhead box P3 (FoxP3) (CST; D608R). Envision HRP-linked polymer (Dako, K4001) and 3,3′-diaminobenzidine (Dako, K3468) were used for visualization of bound primary antibodies. Tissue sections were counterstained with hematoxylin. For sequential double staining, we have used primary antibodies such as QUIN (ImmuSmol; IS1010), NeuN (Abcam; Ab177487), MBP (Abcam, ab40390), GFAP (DAKO; Z0334), and IBA1 (Wako; 019-19741) after epitope retrieval. Bond Polymer Refine Detection (Leica; DS9800) and Bond Polymer Refine Red Detection (Leica; DS9390) were used for visualization of bound primary antibodies. Further tissue sections were counterstained with methyl green. Sections treated with isotype control and without primary antibody were kept as negative controls.

### Statistical analysis

All statistical analyses were performed using GraphPad Prism. Results were expressed as mean ± SD. For clinical scores, significance between each two groups was examined by using multiple range analysis of variance test for multiple comparisons. For RT-PCR, GCMS, and HPLC, we used one-way or two-way ANOVA to identify differences between groups. Data was checked for normality and equal variances between groups. Differences among groups were considered significant if *p* < 0.05. The linearity of the correlation of plasma and CNS samples was tested with Pearson correlation. The required sample size was calculated based on the similar experiments and analyses carried out previously. The number of animals in each experiment is stated in the respective figure legends.

## Results

### EAE-induced mice exhibited clinical signs and displayed inflammation and demyelination

To analyze the KP (Fig. 1a) in EAE mice, C57BL/6 mice were injected with MOG peptide. MOG induced EAE mice exhibited clinical signs between 9 and 14 days (Fig. 1b). Disease severity was compared to healthy animals (Fig. 1c; *p* ≤ 0.001). Signs indicated paralytic EAE exacerbation with simultaneous loss of body weight (Fig. 1d), a characteristic feature of EAE that is correlated with disease severity [17, 18]. Pathological analysis of CNS tissue showed that the EAE group displayed inflammation (Fig. 1e–g), demyelination (Fig. 1h–j), and neuronal loss (Fig. 1k–m) compared to the healthy group.

### The KP is activated in EAE mice and correlates with severity score

**Fig. 2:**
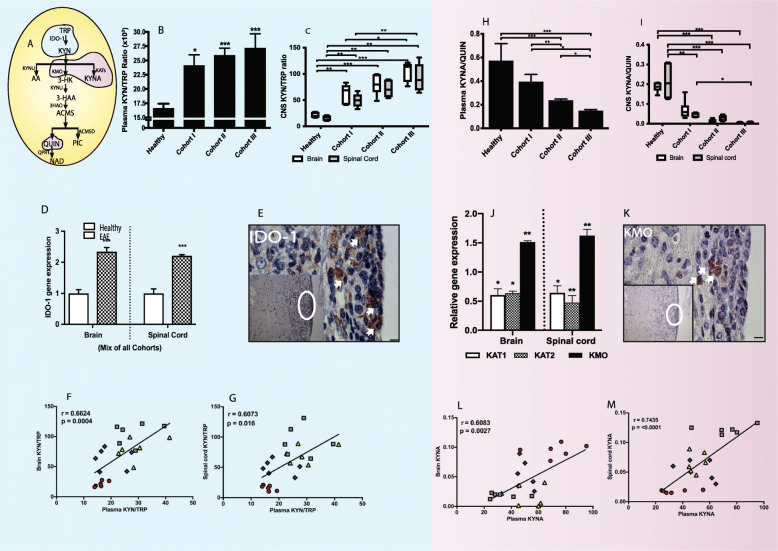
Activation of KP and divergence towards neurotoxicity in EAE (experimental autoimmune encephalomyelitis) mice disease progression. **a** Schematic illustration of kynurenine pathway (KP). **b** and **c** The bar diagrams depict kynurenine (KYN) and tryptophan (TRP) ratio (KYN/TRP) (mean ± SD) measured in the plasma (**b**), brain (white), and spinal cord (gray) (**c**) in healthy (*n* = 8) and EAE cohorts (*n* = 6 per cohort; EAE score of cohort I, 0.5–1; II, 1.5–2; III, 3–4). **d** The histogram depicts IDO-1 gene expression in the brain and spinal cord of healthy (clear) and EAE-induced (filled) mice. (E) The representative lumbar spinal cord of cohort III EAE mice (*n* = 2) section stained with IDO-1 protein (white arrow-brown DAB staining). Scale, × 100 and × 20 (inset). **f** and **g** The graphs represent relationship of KYN/TRP between plasma and CNS tissues (brain (**f**) and spinal cord (**g**)). **h** and **i** The bar diagrams depict kynurenic acid (KYNA) and quinolinic acid (QUIN) ratio (KYNA/QUIN) (mean ± SD) measured in the plasma (**h**), brain (white), and spinal cord (gray) (**i**) in healthy (*n* = 8) and EAE cohorts (*n* = 6 per cohort; EAE score of cohort I, 0.5–1; II, 1.5–2; III, 3–4). **j** The bar diagrams depict relative KAT1 (white), KAT2 (pattern), and KMO (black) gene expressions in the brain and spinal cord of EAE-induced mice relative to healthy control. **k** The representative lumbar spinal cord of cohort III EAE mice (*n* = 2) section stained with KMO protein (white arrow-brown DAB staining). Scale × 100 and × 20 (inset). **l** and **m** The graphs represent relationship of KYN/TRP between plasma and CNS tissues (brain (**l**) and spinal cord (**m**)). ***p ≤ 0.001; **p ≤ 0.01; *p ≤ 0.05. IDO-1, indoleamine 2,3-dioxygenase; KAT, kynurenine amino transferase; KMO, kynurenine monooxygenase; KYNU, kynureninase; 3HAO, 3-hydroxyanthranilate 3,4-dioxygenase; ACMSD, 2-amino-3-carboxymuconatesemialdehyde decarboxylase; QPRT, quinolinate phosphoribosyl transferase

The IDO-1 activity was calculated using TRP and KYN concentrations, expressed as the KYN/TRP ratio (Fig. 2a) compared across different clinical score groups and healthy control. The KYN/TRP ratio was significantly increased in the plasma (*p* < 0.05), brain (*p* < 0.01), and spinal cord (*p* < 0.01) of EAE mice compared to the control group, confirming activation of the KP in EAE progression (Fig. 2b, c). We also observed a significant difference in IDO-1 activation between the least severe group (cohort I) and the most severe group (cohort III) in both the brain (Fig. 2b; *p* < 0.05) and spinal cord (Fig. 2c; *p* < 0.01). This was further validated using qRT-PCR and immunohistochemistry showing increased *IDO-1* gene expression (Fig. 2d) in CNS tissues and protein expression (Fig. 2e) in the spinal cord of EAE-induced mice. We further analyzed whether the parameters of KP metabolism in the periphery can be used to reflect the observations in the CNS. The KYN/TRP ratio in the plasma showed a strong relationship with the KYN/TRP ratio in both the brain (*r* = 0.6624, *p* = 0.0004; Fig. 2f) and spinal cord (*r* = 0.6073, *p* = 0.016; Fig. 2g) during EAE progression.

### Divergence of the KP towards neurotoxicity

The first branching point of the KP occurs at the KYN level where there are two alternatives: either the production of the neuroprotective compound KYNA or further metabolism leading to downstream products especially QUIN which is neurotoxic (Fig. 2a). In both the CNS tissues and plasma of the EAE mice cohort, there was a significant decrease in KYNA/QUIN ratio in EAE-induced mice (Fig. 2h, i). Within the CNS, the KYNA/QUIN ratio was significantly decreased in the spinal cord tissue of the most severe EAE mice (cohort III) when compared to the early cohort (cohort I) (Fig. 2i; *p* < 0.05). Using qRT-PCR, we also analyzed the expression of the two enzymes, *KAT1* and *2*, involved in the conversion of KYN to KYNA. The expression of both *KAT1* and *2* was significantly downregulated, in both CNS tissues (Fig. 2j; *p* < 0.05) which is in accordance with decreased KYNA in CNS tissues of the EAE cohorts (Fig. 2i; *p* < 0.001). This was further supported by the increased expression of KMO (*p* ≤ 0.01) in both the brain and spinal cord tissues of the mixed cohort (Fig. 2j) and protein expression in the inflamed area of EAE-induced mouse spinal cord showing positive expression of KMO protein (Fig. 2k). Further, plasma KYNA correlated with both the brain and spinal KYNA (*r* = 0.6083 and 0.7435; *p* = 0.0027 and ≤ 0.0001, respectively; Fig. 2l, m).

### The KP profile is associated with decreased neuroprotection and increased neurotoxicity in EAE disease severity

**Fig. 3:**
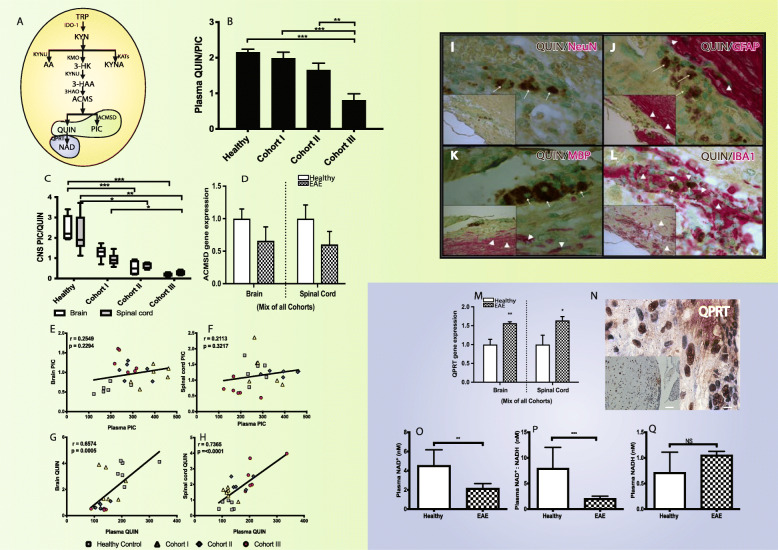
The KP profile is associated with decreased neuroprotection and increased neurotoxicity in EAE (experimental autoimmune encephalomyelitis) disease severity. **a** Schematic illustration of kynurenine pathway (KP). **b** and **c** The bar diagrams depict picolinic acid (PIC) and quinolinic acid (QUIN) ratio (PIC/QUIN) (mean ± SD) measured in the plasma (**b**), brain (white), and spinal cord (gray) in healthy (*n* = 8) and EAE cohorts (*n* = 6 per cohort; EAE score of cohort I, 0.5–1; II, 1.5–2; III, 3–4). **d** The histogram depicts ACMSD gene expression in the brain and spinal cord of healthy (clear) and EAE-induced (filled) mice. **e**–**h** The graphs represent relationship of PIC (**e** and **f**) and QUIN (**g** and **h**) between the plasma vs CNS tissues (brain (**e** and **g**) or spinal cord (**f** and **h**)). **i**–**l** The immunohistochemical staining of lumbar spinal cord of cohort III EAE mice sections showed expression of quinolinic acid (QUIN) with neuron (NeuN) (**i**), QUIN with astrocytes (GFAP) (**j**), QUIN with oligodendrocytes (**k**), and QUIN with monocyte lineage cells (**l**). Arrows show QUIN staining (brown), and arrow heads show neuronal and glial markers staining (red). Counterstain, methyl green. Scale bars 10 um; 5 um (inset). **m** The bar diagram depicts QPRT gene expression in the brain and spinal cord of healthy (clear) and EAE-induced (filled) mice. **n** The immunohistochemical staining of lumbar spinal cord of cohort III EAE mice sections showed expression of QPRT protein (*n* = 2). Scale × 100 and × 20 (inset). **o**–**q** The bar diagrams represent serum NAD+ (nicotinamide adenine dinucleotide (oxidized)) (**o**), NADH (nicotinamide adenine dinucleotide (reduced)) (**p**), and NAD+:NADH ratio (**q**) in healthy and EAE mice (mean ± SD). ***p ≤ 0.001; **p ≤ 0.01; *p ≤ 0.05. IDO-1, indoleamine 2,3- dioxygenase; KAT, kynurenine amino transferase; KMO, kynurenine monooxygenase; KYNU, kynureninase; 3HAO, 3-hydroxyanthranilate 3,4-dioxygenase; ACMSD, 2-amino-3- carboxymuconate-semialdehyde decarboxylase; QPRT, quinolinate phosphoribosyl transferase

The last branching point within the KP occurs at *ACMSD* where either PIC (through ACMSD) or QUIN (non-enzymatically) is produced (Fig. 3a). The PIC/QUIN ratio was significantly decreased in both the periphery (Fig. 3b) and CNS (Fig. 3c) of cohort III EAE induced compared to controls. There was a significant (*p* ≤ 0.001) progressive reduction of PIC/QUIN ratio in the CNS from cohorts I to III. This effect was more apparent with significantly (*p* ≤ 0.001) increasing QUIN production as the disease severity increased (i.e., from cohorts I to III) (Fig. 3c). Furthermore, expression of the *ACMSD* gene was downregulated (Fig. 3d) in comparison to the control, indicating that the pathway was directed to synthesize more of the excitotoxin QUIN at the expense of PIC production. We did not observe any correlation for PIC concentrations between periphery and CNS (*r* = 0.2549 and 0.2113; for the brain and spinal cord, respectively; Fig. 3e, f). However, we observed that plasma QUIN highly correlated with both the brain and spinal cord QUIN concentration (*r* = 0.6574 and 0.7365; *p* = 0.0005 and < 0.0001, respectively; Fig. 3g, h).

### Increased QUIN production by monocyte lineage cells

To assess the mechanistic link between cell types and QUIN synthesis, we performed double staining on the demyelinated lumbar section of EAE cohort III mice spinal cord with marker combinations such as QUIN/NeuN (neuron), QUIN/GFAP (astrocyte), QUIN/IBA1 (macrophage/microglia), and QUIN/MBP (oligodendrocytes) and counterstained with methyl green. We have found almost no QUIN production in neurons (NeuN) (Fig. 3i), astrocytes (GFAP) (Fig. 3j), or oligodendrocytes (MBP) (Fig. 3k). Macrophage/microglial cells strongly colocalized with QUIN (Fig. 3l).

### Chronic accumulation of QUIN

QUIN is catabolized by the enzyme QPRTase to synthesize NAD^+^ (Fig. 3a). Notably, we found that *QPRT* was significantly upregulated, indicating increased activity in the catabolism of QUIN in EAE mice (Fig. 3m; *p* < 0.05). QPRT protein expression was also observed in the inflamed areas of EAE-induced mice (Fig. 3n). Despite *QPRT* upregulation, QUIN was significantly increased (cohort III: *p* ≤ 0.001) in both the CNS and periphery (plasma) of EAE-induced mice in comparison to healthy controls (Fig. 3b, c). In order to test whether increased *QPRT* activity was associated with increased NAD^+^ production, we measured the NAD^+^ and NADH in EAE samples. We observed a significant decrease in plasma NAD^+^ (*p* = 0.0079; Fig. 3o) and NAD^+^/NADH ratio (*p* ≤ 0.001; Fig. 3p) in EAE-induced mice compared to healthy controls. However, there was no change in plasma NADH concentration (Fig. 3q).

### KP modulation reverses the EAE severity and increases the neuroprotection

**Fig. 4:**
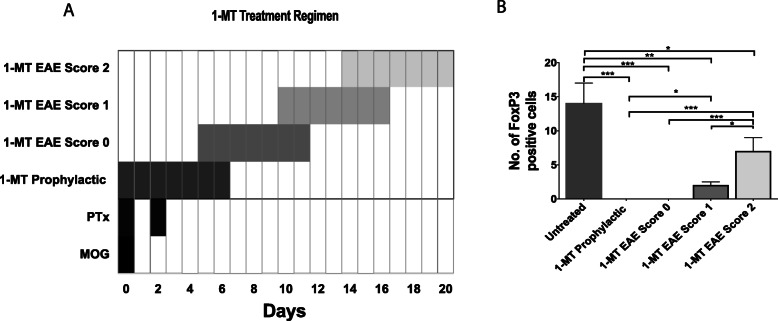
IDO-1 inhibition and immune tolerance. **a** Schematic illustration of indoleamine 2,3 dioxygenase inhibition by 1-methyl-DL-tryptophan (1-MT; 100 mg/kg i.p.; 7 days) at various stages of EAE (experimental autoimmune encephalomyelitis) induction. MOG (myelin oligodendroglial protein) and PTx (pertussis toxin) injected C57BL/6 mice were treated with 1-MT prophylactically or 1-MT treatment after EAE induction at day 5 or 1-MT treatment at EAE clinical score of 1 or 1-MT treatment at EAE clinical score of 2. **b** FoxP3 (forkhead box protein) expression was measured in spleen of 1-MT treated EAE-induced mice (mean ± SD; ***p ≤ 0.001; **p ≤ 0.01; *p ≤ 0.05)

Modulation of the KP at the IDO-1 step by 1-MT in EAE mice showed differential FoxP3 expression depending on the treatment at various stages of the disease (Fig. 4a). Prophylactic 1-MT treatment of mice prior to EAE induction (EAE score 0), 1-MT treatment after EAE induction (day 5: EAE score 0), and 1-MT treatment after the EAE-induced mice reached the score 1 groups showed less or no FoxP3 expression, whereas 1-MT treatment after the EAE-induced mice reached the score 2 was associated with increased FoxP3 expression in the spleen compared to other 1-MT treatment groups (Fig. 4b).

**Fig. 5:**
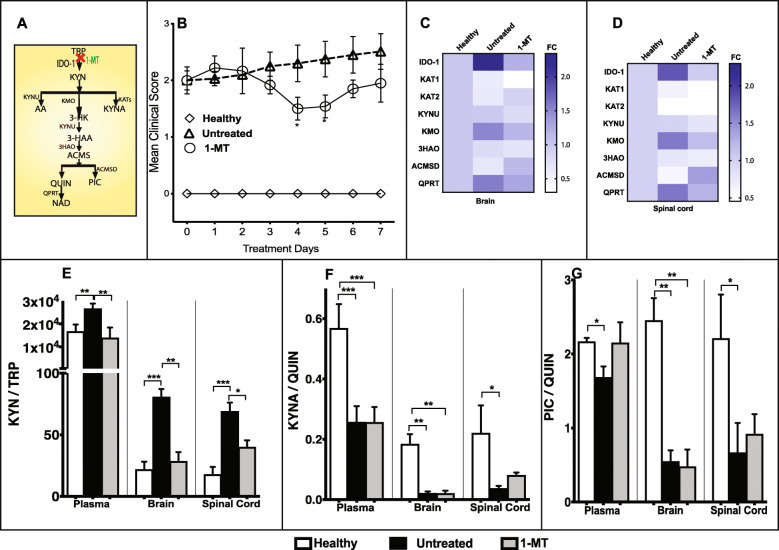
Early modulation of KP ameliorates the progression of EAE (experimental autoimmune encephalomyelitis). **a** Schematic illustration of kynurenine pathway (KP). **b** Mean EAE clinical scores of healthy control (◊, *n* = 6), untreated EAE control (Δ, *n* = 6), and 1methyl-DL-tryptophan treated EAE mice (1-MT; IDO-1 inhibitor; O; *n* = 6, 100 mg/kg; i.p. daily for 7 days) (mean ± SD, *p < 0.05 (1-MT vs untreated EAE)). **c** and **d** The heat map shows KP enzyme gene expression in the brain (**c**) and spinal cord (**d**) of healthy, untreated, and 1-MT treated EAE mice. Gene expression ranges from high (dark blue) to low (white). **e**–**g** Changes in KP metabolites upon treatment with 1-MT. The bar diagrams depict kynurenine and tryptophan (KYN/TRP) ratio (**e**), kynurenic acid, and quinolinic acid (KYNA/QUIN) ratio (**f**), and picolinic acid and quinolinic acid (PIC/QUIN) ratio (**g**) measured (mean ± SD) in the plasma, brain, and spinal cord of healthy (white; *n* =8), untreated (black; *n* = 6), and 1-MT (gray; *n* = 3) treated mice. Three symbols: *p* ≤ 0.001; two: *p* ≤ 0.01; one: *p* ≤ 0.05. IDO-1, indoleamine 2,3-dioxygenase; KAT, kynurenine amino transferase; KMO, kynurenine monooxygenase; KYNU, kynureninase; 3HAO, 3-hydroxyanthranilate 3,4-dioxygenase; ACMSD, 2-amino-3-carboxymuconatesemialdehyde decarboxylase; QPRT, quinolinate phosphoribosyl transferase

1-MT treatment (Fig. 5a) after the EAE-induced mice reached the score of 2 has shown significant reduction in clinical severity (EAE score, 2.5 to 1.5; *p* < 0.05) compared to untreated control (Fig. 5b). EAE-induced mice with score of 2 treated with 1-MT significantly inhibited the IDO-1 gene expression (*p* ≤ 0.001 (brain; Fig. 5c) and ≤ 0.05 (spinal cord; Fig. 5d)). Expression of *KAT1* enzyme was significantly reduced (*p* ≤ 0.01 (brain; Fig. 5c) and ≤ 0.05 (spinal cord; Fig. 5d)) in EAE mice treated with 1-MT after score of 2. However, *KAT2* gene expression did not show any significant change in CNS tissues (Fig. 5c, d) in this group. Further, there was significant downregulation in IDO-1 activity in periphery and CNS tissues (Fig. 5e) upon treatment with 1-MT in EAE-induced mice (score 2). However, no significant differences in KYNA/QUIN (Fig. 5f) and PIC/QUIN (Fig. 5g) ratios were observed in 1-MT treated mice compared to untreated controls.

**Fig. 6:**
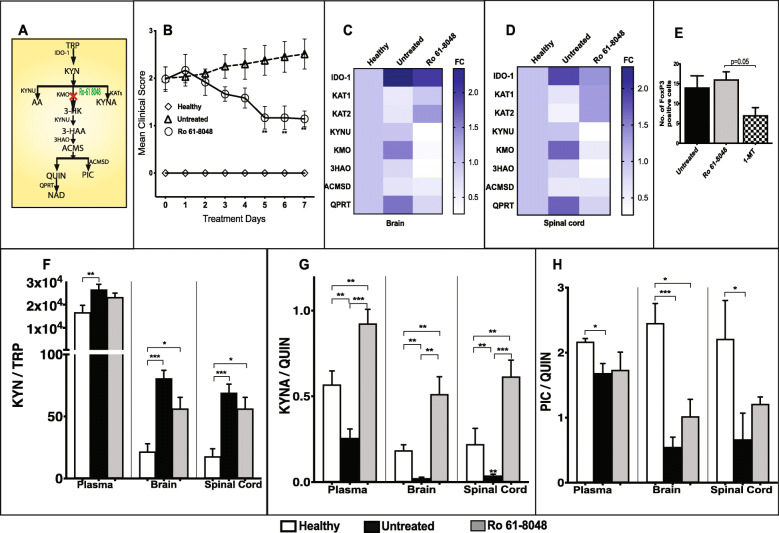
Delayed modulation of KP reverses the progression of experimental autoimmune encephalomyelitis (EAE). **a** Schematic illustration of kynurenine pathway (KP). **b** Mean EAE clinical scores of healthy control (◊, *n* = 6), untreated EAE control (Δ, *n* = 6), and Ro 61-8048 treated EAE mice (KMO inhibitor; O; *n* = 6), 100 mg/kg; i.p. daily for 7 days (mean ± SD, *p < 0.05 (Ro 618048 vs untreated EAE)). **c** and **d** The heat maps show KP gene expression in the brain (**c**) and spinal cord (**d**) of healthy, untreated and Ro 61 8048 treated EAE mice. Gene expression ranges from high (dark blue) to low (white). **e** FoxP3 (forkhead box protein) expression was measured in spleen of 1-MT and Ro 61-8048 treated EAE-induced mice (EAE score 2). **f**–**h** Changes in KP metabolites upon treatment with Ro 61-8048. The line diagrams depict kynurenine and tryptophan (KYN/TRP) ratio (**f**), kynurenic acid, and quinolinic acid (KYNA/QUIN) ratio (**g**), and picolinic acid and quinolinic acid (PIC/QUIN) ratio (**h**) measured (mean ± SD) in the plasma, brain, and spinal cord of healthy (white; *n* = 8), untreated EAE (black; *n* = 6), and Ro 61-8048 (gray; *n* = 3) treated mice. Three symbols: *p* ≤ 0.001; two: *p* ≤ 0.01; one: *p* ≤ 0.05. IDO-1, indoleamine 2,3-dioxygenase; KAT, kynurenine amino transferase; KYNU, kynureninase; KMO, kynurenine monooxygenase; 3HAO, 3-hydroxyanthranilate 3,4-dioxygenase; ACMSD, 2-amino-3-carboxymuconatesemialdehyde decarboxylase; QPRT, quinolinate phosphoribosyl transferase

Later modulation of the KP, once the EAE score was 2, at the KMO step by Ro 61-8048 (Fig. 6a) also significantly reduced the clinical severity (EAE score 2.5 to 1; *p* ≤ 0.01) (Fig. 6b), which was associated with significant decrease in *KMO* gene expression (Fig. 6c, d) in CNS tissues. Further, there was increased *KAT1* and *KAT2* gene expression especially in the mixed cohort of spinal cord tissues of the Ro 61-8048 treated group (Fig. 6d). Upon KMO inhibition, decreased *KYNU* (*p* ≤ 0.05), *3HAO* (*p* > 0.05), and *QPRT* gene expression (*p* ≤ 0.05) were observed in the Ro 61-8048 treated group compared to untreated controls (Fig. 6c, d). Further, the Ro 61-8048 treated group was associated with significantly greater FoxP3 expression (Fig. 6e; *p* = 0.05) compared to the 1-MT treated group at score 2. Metabolite analysis revealed no significant difference in KYN/TRP (Fig. 6f) and PIC/QUIN (Fig. 5h) in treated versus untreated groups in both periphery and CNS tissues but showed a significant increase in KYNA/QUIN ratio (Fig. 6g) in both plasma and CNS tissues (*p* < 0.001).

## Discussion

The KP is activated in our EAE model in response to inflammatory cytokines and chemokines [19]. This initial response is protective by increasing immune tolerance. However, the long-term activation of the KP generates an environment in which ongoing production of the toxic KP metabolite, QUIN, contributes to the chronic inflammatory and neurotoxic landscape associated with neurodegeneration. In this context, QUIN contributes to bridging the neuroinflammation and neurodegeneration aspects of EAE with direct implication for MS [20, 21]. The balance between neurotoxicity and neuroprotection is critical for the outcome of KP activation and intervention. We showed that treatment intervention after the disease score of 2 with KP modulators is associated with significant beneficial effects compared to early intervention in our MS model. Early IDO-1 activation, instigating immune tolerance and reduced neurotoxic to neuroprotective ratio contributes to the beneficial effects of KP modulation. When taken with our recently published findings of aberrant KP metabolomics in MS patients [9], the current results have direct relevance to MS pathogenesis and treatment.

During EAE progression, our data shows a shift from neuroprotective to neurotoxic KP metabolites over time. The relative concentration of the KYNA/QUIN and PIC/QUIN favors QUIN accumulation. QUIN is proinflammatory, neurotoxic, and gliotoxic acting through several different mechanisms [10]. We also showed that QUIN was elevated in the mice plasma and CNS tissues. While previous studies have shown that to antagonize QUIN excitotoxicity, endogenous KYNA levels have to be approximately twice the amount of QUIN [22]; this was not the case in our current study, nor in our published MS study [7, 8]. In our in vivo study, QUIN concentrations reached approximately 4 times the normal levels from 0.662 ± 0.11 nM/g (healthy control) to 2.625 ± 0.406 nM/g (EAE cohort III) at which level toxicity is known to occur, whereas KYNA levels were only 0.15 times (0.122 ± 0.003 nM/g (healthy control) and 0.018 ± 0.001 nM/g (EAE cohort III)) in the spinal cord. Further, in agreement with our previous in vitro study [23], our in vivo results showed that almost all QUIN expressing cells are macrophage/microglial cells but not neurons, astrocytes, or oligodendrocytes. This shows that chronic inflammatory responses might easily trigger cumulative production of pathophysiological concentrations of QUIN by activated monocytic cells such as infiltrating macrophages and microglia. We previously showed that activated monocytic cells produce pathophysiological concentrations of QUIN able to induce both neuronal and glial cell death [24]. The importance of monocytes in MS is being increasingly appreciated, as they are the most abundant cell type in the CSF and plaques, implying that monocyte entry is a key event in MS [25]. Further, monocytes are directly associated with oligodendrocytes and importantly, initiated the demyelination [26]. This likely creates an environment in which other neuronal cells are highly susceptible to excitotoxicity.

We consider that our results point to significantly less neurodegeneration with appropriately timed KP inhibition for the following reasons. We showed that the levels of the excitotoxin QUIN (produced from the KP by activated monocytic cells) significantly increased with EAE disease stages (Fig. 3) and significantly dropped with KP inhibition with concordant less central nervous system damage. Second, we have previously shown the link between the KP and the switch to neurodegeneration in progressive MS [9]. Third, the product of the KP, QUIN, can kill neurons [27], astrocytes [20], and oligodendrocytes [15] at nanomolar concentrations (as seen in chronic neurodegenerative conditions) and increase the levels of several proinflammatory cytokines and chemokines [20] that have been linked to various neurodegenerative conditions. Fourth, KP metabolites correlate with several neurodegenerative diseases such as Huntington’s disease, Alzheimer’s disease, and Parkinson’s disease.

Interestingly, we found that the enzyme *QPRT* was highly expressed in EAE mice compared to controls. In such instances, one should observe a decrease in QUIN production, as this enzyme is responsible for the catabolism of QUIN to NAD^+^. However, in EAE-induced mice, the QUIN concentrations remain high in both periphery and CNS because the QPRT enzyme is saturable at low QUIN concentrations (~ 300 nM) [27]. Further, total tissue NAD^+^ levels were significantly decreased in EAE-induced mice compared to controls because of cell dysfunction and loss. In EAE, it was reported that nicotinamide, a precursor of NAD^+^ biosynthesis [28] and NAD^+^ [18], reduced the CD4+ T-cell infiltration and differentiation, respectively, and demyelination. In contrast, a different study found that inhibition of NAD+ synthesis by FK866, an inhibitor of nicotinamide phosphoribosyltransferase (Nampt), protected against EAE by depleting NAD^+^ in activated CD3+ T lymphocytes and impairing their proliferation and cytokine production [29]. In our study, decreased NAD^+^ could represent a self-protective mechanism by decreasing the NAD+ availability for T-cells in EAE mice. An additional possible mechanism at the cellular level is that persistent NAD-induced activation of poly (ADP-ribose) polymerase (PARP) is known to lead to NAD depletion [30]. The PARP family of enzymes, particularly PARP-1, is DNA binding enzymes, activated by free-radical mediated DNA strand breaks that are involved in DNA repair and maintenance of genomic integrity. PARP uses NAD^+^ to make ADP ribose polymers. An increase in DNA damage, often because of oxidative stress, can rapidly deplete the cell of NAD^+^ resulting in reduced ATP production and cell death [31]. Our recent data has also shown that chronic KMO activation of the KP leads to significant oxidative stress [32].

We previously found that low NAD^+^ in MS patients correlates with severity and progression of the disease [33]. Thus, QUIN is chronically accumulating by infiltrating microglial/macrophage cells as EAE progresses and is a likely contributor to the neurodegeneration with reduced de novo NAD^+^ synthesis. Based on the above findings, high QUIN levels in EAE mouse model could represent one of the key factors in linking neuroinflammation and neurodegeneration.

The results of our study with KP modulation are promising. Inhibition of IDO-1 with 1-MT after the initiation of immune tolerance (score 2) significantly decreases the clinical severity compared to untreated controls. However, previous studies suggested that activation of IDO-1 alleviated the disease symptoms [11, 34, 35]. Kwidzinski et al. [36] showed that the administration of the IDO inhibitor, 1-MT, exacerbated the condition when given at 50 mg/ml, subcutaneously. This discrepancy in the results is likely associated with the time of administration of the drug. Previous studies administered the 1-MT prior to EAE induction or during the EAE induction procedure [11, 34, 35]. Previous studies have shown that the 1-MT effect that had inhibited the KP at an earlier time point before the development of tolerance. In our study, we compared the 1-MT administration at various disease stages. Only 1-MT treatment after the score of 2 in EAE mice showed significantly improved the FoxP3 expression compared to other treatment groups. FoxP3 is a master regulator of the regulatory pathway in the development and function of regulatory T-cells involved in immune tolerance. Decreased clinical severity is associated with earlier IDO-1 activation to trigger immune tolerance (FoxP3) and also therapeutic ability of 1-MT to decrease formation and accumulation of QUIN. Also, treatment with 1-MT after EAE-induced mice that reached the score of 2 (therapeutic approach) is similar to human patients, who receive treatment after the onset of the disease. This shows that the expression of IDO-1 is a preventative mechanism in EAE and illustrates the importance of dampening the inflammation by *short-term* IDO-1 activation. However, *prolonged* IDO-1 activation was shown to cumulatively increase the neurotoxicity [11, 37]. This could be prevented by timed administration of KP modulator, 1-MT.

Platten et al. [38] tested the synthetic anthranilic acid derivative (Tranilast), a downstream KP metabolite in relapsing-remitting EAE model. They showed that the decreased disease severity was associated with the inhibition of the Th1 cells by inducing antigen-specific IL-10 producing T-cells with regulatory potential [38]. These increased immunosuppressive IL-10 producing T-cells might be because of excessive NAD^+^ production through more substrate influx [18]. Recently, Mondanelli et al. [35] showed that the N-acetylserotonin acts as positive allosteric modulator of the IDO-1 enzyme and protects the EAE mice from neuroinflammation by increasing the kynurenine-mediated AhR activation. Also, there was increased IL-10 and reduced the secretion of IL-17A and IFN-γ in NAS treated EAE mice. However, these authors did not measure the downstream catabolites such as QUIN, NAD^+^, and PIC. Although QUIN is neurotoxic [39], the QUIN by-product NAD^+^ released during inflammation takes part in the Treg homeostasis in vivo through an ART2.2-dependent P2X7-mediated mechanism (NAD induced cell death) [40].

In this regard, KMO is particularly interesting as its position is at the branching point in the KP between KYNA and 3-HK synthesis. Thus, inhibition of KMO is predicted to increase the flux through the neuroprotective KYNA branch, thereby reducing the flux through the neurotoxic branch of 3-HK and QUIN, ultimately resulting in neuroprotection [41]. The study by Chiarugi et al. supports our findings, and we further provide evidence that KMO inhibition is associated with increased KYNA synthesis. KMO is predominantly expressed in microglia [42, 43].

Several studies indicate that the innate immunity drives the progressive stage of MS involving activated microglia and infiltrating macrophages [44]. During prolonged activation of microglia and macrophages, large amounts of KYN produced by astrocytes can be used as a substrate for production of QUIN through KP activation [19]. QUIN will also turn astrocytes to a proinflammatory phenotype with decreased biosynthesis of KYNA and increased production of inflammatory mediators [45]. When we inhibited KMO using Ro 61-8048, as predicted, there was an increase in KYNA in both the brain and spinal cord. Similar findings have been reported in animal models of cerebral ischemia with Ro 61-8048 treatment [46] and MBP induced EAE rats [47].

In EAE-induced mice, the KP biomarkers in plasma samples correlated well with CNS tissue samples. This is in accordance with our recent study in human MS samples [9] and confirms the feasibility of a blood-based KP biomarker for MS to signal the development of neurodegeneration as part of the transition to the secondary progressive phase of MS. There is another important implication of our findings. Human trials with IDO-1 inhibitors as cancer therapies have been disappointing without a clear reason. Our data points to the efficacy of modulation of the KP rather than inhibition. Modulation has to be appropriately “timed” in relation to the particular pathogenetic steps of the disease, and efficacy for appropriately timed KP modulation is best at the KMO step. These results are likely to be applicable to other disorders where the KP is activated. Furthermore, they provide impetus to the development of monocyte/macrophage directed therapies utilizing KP modulation for MS and other diseases where the monocyte/macrophage have significant pathogenetic functions. This would complement existing T- and B-cell-directed therapies given that the KP is not present in lymphocytes [48].

## Conclusion

In conclusion, this study provides strong evidence of the KP involvement in EAE disease pathogenesis, particularly in disease progression and severity. Also, these data provide solid support for appropriately timed KP modulation as part of MS treatment to slow and possibly halt disease progression.

## Data Availability

The data that supports the findings of this study is available from the corresponding author upon reasonable request.

## Abbreviations

EAE: Experimental autoimmune encephalomyelitis
KMO: Kynurenine monooxygenase
KP: Kynurenine pathway
KYN: Kynurenine
KYNA: Kynurenic acid
MS: Multiple sclerosis
NAD^+^: Oxidized nicotinamide adenine dinucleotide
NADH: Reduced nicotinamide adenine dinucleotide
PIC: Picolinic acid
QUIN: Quinolinic acid
TRP: Tryptophan
IDO-1: Indoleamine 2,3-dioxygenase
KAT: Kynurenine amino transferase
KMO: Kynurenine monooxygenase
KYNU: Kynureninase
3HAO: 3-Hydroxyanthranilate 3,4-Dioxygenase
ACMSD: 2-Amino-3-carboxymuconate-semialdehyde decarboxylase
QPRT: Quinolinate phosphoribosyl transferase
FoxP3: Forkhead box P3

## References

[1] Sospedra M, Martin R (2005). Immunology of multiple sclerosis. Ann Rev Immunol..

[2] Crutcher KA, Gendelman HE, Kipnis J, Perez-Polo JR, Perry VH, Popovich PG (2006). Debate: “is increasing neuroinflammation beneficial for neural repair?”. J Neuroimmune Pharmacol..

[3] Imam SA, Guyton MK, Haque A, Vandenbark A, Tyor WR, Ray SK (2007). Increased calpain correlates with Th1 cytokine profile in PBMCs from MS patients. J Neuroimmunol\..

[4] Munn DH, Mellor AL (2016). IDO in the tumor microenvironment: inflammation, counter-regulation, and tolerance. Trends Immunol..

[5] Monaco F, Fumero S, Mondino A, Mutani R (1979). Plasma and cerebrospinal fluid tryptophan in multiple sclerosis and degenerative diseases. J Neurol Neurosurg Psychiatry..

[6] Mellor AL, Munn DH (2004). IDO expression by dendritic cells: tolerance and tryptophan catabolism. Nature Rev Immunol..

[7] Hartai Z, Klivenyi P, Janaky T, Penke B, Dux L, Vecsei L (2005). Kynurenine metabolism in multiple sclerosis. Acta Neurologica Scandinavica..

[8] Rejdak K, Bartosik-Psujek H, Dobosz B, Kocki T, Grieb P, Giovannoni G (2002). Decreased level of kynurenic acid in cerebrospinal fluid of relapsing-onset multiple sclerosis patients. Neuroscience letters..

[9] Lim CK, Bilgin A, Lovejoy DB, Tan V, Bustamante S, Taylor BV (2017). Kynurenine pathway metabolomics predicts and provides mechanistic insight into multiple sclerosis progression. Sci Rep..

[10] Guillemin GJ, Kerr SJ, Brew BJ (2005). Involvement of quinolinic acid in AIDS dementia complex. Neurotoxicity Res..

[11] Sakurai K, Zou JP, Tschetter JR, Ward JM, Shearer GM (2002). Effect of indoleamine 2,3-dioxygenase on induction of experimental autoimmune encephalomyelitis. Journal of neuroimmunology..

[12] Rodgers J, Stone TW, Barrett MP, Bradley B, Kennedy PG (2009). Kynurenine pathway inhibition reduces central nervous system inflammation in a model of human African trypanosomiasis. Brain..

[13] Brown DA, Sawchenko PE (2007). Time course and distribution of inflammatory and neurodegenerative events suggest structural bases for the pathogenesis of experimental autoimmune encephalomyelitis. J Comp Neurol.

[14] Racke MK. Experimental autoimmune encephalomyelitis (EAE). Curr Protoc Neurosci. 2001; Chapter 9:Unit 9.7. 10.1002/0471142301.ns0907s14 18428555

[15] Sundaram G, Brew BJ, Jones SP, Adams S, Lim CK, Guillemin GJ. Quinolinic acid toxicity on oligodendroglial cells: relevance for multiple sclerosis and therapeutic strategies. J Neuroinflammation. 2014;11(204). 10.1186/s12974-014-0204-5 PMC4302518 25498310

[16] Pfaffl MW (2001). A new mathematical model for relative quantification in real-time RT-PCR. Nucleic Acids Res.

[17] Miller SD, Karpus WJ, Davidson TS (2010). Experimental autoimmune encephalomyelitis in the mouse. Curr Protocols Immunol..

[18] Tullius SG, Biefer HR, Li S, Trachtenberg AJ, Edtinger K, Quante M (2014). NAD+ protects against EAE by regulating CD4+ T-cell differentiation. Nat Commun..

[19] Guillemin GJ, Kerr SJ, Pemberton LA, Smith DG, Smythe GA, Armati PJ (2001). IFN-beta1b induces kynurenine pathway metabolism in human macrophages: potential implications for multiple sclerosis treatment. J Interferon Cytokine Res..

[20] Guillemin GJ (2012). Quinolinic acid, the inescapable neurotoxin. The FEBS journal..

[21] Lovelace MD, Varney B, Sundaram G, Franco NF, Ng ML, Pai S (2016). Current evidence for a role of the kynurenine pathway of tryptophan metabolism in multiple sclerosis. Front Immunol..

[22] Lekieffre D, Plotkine M, Allix M, Boulu RG (1990). Kynurenic acid antagonizes hippocampal quinolinic acid neurotoxicity: behavioral and histological evaluation. Neuroscience letters..

[23] Guillemin GJ, Smythe G, Takikawa O, Brew BJ (2005). Expression of indoleamine 2,3-dioxygenase and production of quinolinic acid by human microglia, astrocytes, and neurons. Glia..

[24] Guillemin GJ, Kerr SJ, Smythe GA, Smith DG, Kapoor V, Armati PJ (2001). Kynurenine pathway metabolism in human astrocytes: a paradox for neuronal protection. J Neurochem..

[25] Waschbisch A, Schroder S, Schraudner D, Sammet L, Weksler B, Melms A (2016). Pivotal role for CD16+ monocytes in immune surveillance of the central nervous system. J Immunol..

[26] Yamasaki R, Lu H, Butovsky O, Ohno N, Rietsch AM, Cialic R (2014). Differential roles of microglia and monocytes in the inflamed central nervous system. J Exp Med..

[27] Rahman A, Ting K, Cullen KM, Braidy N, Brew BJ, Guillemin GJ (2009). The excitotoxin quinolinic acid induces tau phosphorylation in human neurons. PloS one..

[28] Kaneko S, Wang J, Kaneko M, Yiu G, Hurrell JM, Chitnis T (2006). Protecting axonal degeneration by increasing nicotinamide adenine dinucleotide levels in experimental autoimmune encephalomyelitis models. J Neurosci.

[29] Bruzzone S, Fruscione F, Morando S, Ferrando T, Poggi A, Garuti A (2009). Catastrophic NAD+ depletion in activated T lymphocytes through Nampt inhibition reduces demyelination and disability in EAE. PloS one..

[30] Braidy N, Grant R, Adams S, Brew BJ, Guillemin GJ (2009). Mechanism for quinolinic acid cytotoxicity in human astrocytes and neurons. Neurotoxicity Res..

[31] Braidy N, Guillemin G, Grant R (2008). Promotion of cellular NAD(+) anabolism: therapeutic potential for oxidative stress in ageing and Alzheimer’s disease. Neurotoxicity Res..

[32] Castellano-Gonzalez G, Jacobs KR, Don E, Cole NJ, Adams S, Lim CK, et al. Kynurenine 3-monooxygenase activity in human primary neurons and effect on cellular bioenergetics identifies new neurotoxic mechanisms. Neurotoxicity Res. 2019. 10.1007/s12640-019-9997-4 30666558

[33] Braidy N, Lim CK, Grant R, Brew BJ, Guillemin GJ (2013). Serum nicotinamide adenine dinucleotide levels through disease course in multiple sclerosis. Brain Res..

[34] Xiao BG, Wu XC, Yang JS, Xu LY, Liu X, Huang YM (2004). Therapeutic potential of IFN-gamma-modified dendritic cells in acute and chronic experimental allergic encephalomyelitis. International immunology..

[35] Mondanelli G, Coletti A, Greco FA, Pallotta MT, Orabona C, Iacono A, et al. Positive allosteric modulation of indoleamine 2,3-dioxygenase 1 restrains neuroinflammation. Proc Natl Acad Sci U S A. 2020. 10.1073/pnas.1918215117 PMC7035626 32024760

[36] Kwidzinski E, Bunse J, Aktas O, Richter D, Mutlu L, Zipp F (2005). Indolamine 2,3-dioxygenase is expressed in the CNS and down-regulates autoimmune inflammation. FASEB J.

[37] Matysiak M, Stasiolek M, Orlowski W, Jurewicz A, Janczar S, Raine CS (2008). Stem cells ameliorate EAE via an indoleamine 2,3-dioxygenase (IDO) mechanism. J Neuroimmunol..

[38] Platten M, Ho PP, Youssef S, Fontoura P, Garren H, Hur EM (2005). Treatment of autoimmune neuroinflammation with a synthetic tryptophan metabolite. Science..

[39] el-Defrawy SR, Boegman RJ, Jhamandas K, Beninger RJ. The neurotoxic actions of quinolinic acid in the central nervous system. Can J Physiol Pharmacol. 1986;64(3):369-375. 10.1139/y86-060 2939936

[40] Hubert S, Rissiek B, Klages K, Huehn J, Sparwasser T, Haag F (2010). Extracellular NAD+ shapes the Foxp3+ regulatory T cell compartment through the ART2-P2X7 pathway. J Exp Med..

[41] Thevandavakkam MA, Schwarcz R, Muchowski PJ, Giorgini F (2010). Targeting kynurenine 3-monooxygenase (KMO): implications for therapy in Huntington’s disease. CNS Neurol Disord Drug Targets..

[42] Heyes MP, Achim CL, Wiley CA, Major EO, Saito K, Markey SP (1996). Human microglia convert l-tryptophan into the neurotoxin quinolinic acid. Biochem J..

[43] Guillemin GJ, Kerr SJ, Smythe GA, Armati PJ, Brew BJ (1999). Kynurenine pathway metabolism in human astrocytes. Adv Exp Med Biol.

[44] Popovich PG, Longbrake EE (2008). Can the immune system be harnessed to repair the CNS?. Nat Rev Neurosci.

[45] Guillemin GJ, Croitoru-Lamoury J, Dormont D, Armati PJ, Brew BJ (2003). Quinolinic acid upregulates chemokine production and chemokine receptor expression in astrocytes. Glia..

[46] Cozzi A, Carpenedo R, Moroni F. Kynurenine hydroxylase inhibitors reduce ischemic brain damage: studies with (m-nitrobenzoyl)-alanine (mNBA) and 3,4-dimethoxy-[-N-4-(nitrophenyl)thiazol-2yl]-benzenesulfonamide (Ro 61-8048) in models of focal or global brain ischemia. Journal of cerebral blood flow and metabolism.1999;19(7):771-777. 10.1097/00004647-199907000-00007 10413032

[47] Chiarugi A, Cozzi A, Ballerini C, Massacesi L, Moroni F (2001). Kynurenine 3-mono-oxygenase activity and neurotoxic kynurenine metabolites increase in the spinal cord of rats with experimental allergic encephalomyelitis. Neuroscience..

[48] Jones SP, Franco NF, Varney B, Sundaram G, Brown DA, de Bie J (2015). Expression of the kynurenine pathway in human peripheral blood mononuclear cells: implications for inflammatory and neurodegenerative disease. PloS one..

